# Effectiveness of acupuncture in migraine rats: A systematic review

**DOI:** 10.1371/journal.pone.0280556

**Published:** 2023-01-20

**Authors:** Pingping Su, Xiuzhen Xie, Yingqi Xu, Xinxin Luo, Jianli Niu, Zhuqing Jin

**Affiliations:** 1 The Third School of Clinical Medicine, Zhejiang Chinese Medical University, Hangzhou, 310053, China; 2 The Second School of Clinical Medicine, Zhejiang Chinese Medical University, Hangzhou, 310053, China; 3 Office of Human Research, Memorial Healthcare System, Hollywood, Florida, United States of America; 4 School of Basic Medical Sciences, Zhejiang Chinese Medical University, Hangzhou, 310053, China; UCSI: UCSI University, MALAYSIA

## Abstract

**Objective:**

To systematically evaluate the effectiveness and potential underlying mechanisms of acupuncture in the treatment of experimental model of migraine in rats.

**Methods:**

Nine electronic databases, including CNKI (China National Knowledge Infrastructure), WanFang, VIP (Chinese Scientific Journals Database), Sinomed, PubMed, Cochrane Library, Web of Science and EBSCO, were searched for randomized experimental studies on migraine in rats involving acupuncture intervention. The search period ranged from inception to June 2022. The methodological quality was assessed using the SYRCLE’s risk of bias tool for animal studies. Data were analyzed using the Revman 5.3 software.

**Results:**

A total of 13 studies were included in this analysis. Findings from the available experimental studies documented that acupuncture significantly reduced behavior scores of rats with migraine (MD = -15.01, 95%CI = [-18.01, -12.01], *P*<0.00001) and downregulated the expression of calcitonin gene-related peptide (CGRP) (MD = -16.14, 95%CI = [-21.45, -10.83], *P*<0.00001), substance P (SP) (MD = -11.47, 95%CI = [-15.97, -6.98], *P*<0.00001) and nitric oxide (NO) (MD = -3.02, 95%CI = [-3.79, -2.26], *P*<0.00001) in serum, and stimulatory G protein (Gsa) (MD = -62.90, 95%CI = [-69.88, -55.92], *P*<0.00001) in brainstem. Acupuncture also significantly increased the content of inhibitory G protein (Gia) (MD = 24.01, 95%CI = [20.10, 27.92], *P*<0.00001) in brainstem and 50% paw withdrawal threshold (50%PWT) (MD = 1.96, 95%CI = [1.15, 2.77], *P*<0.00001).

**Conclusion:**

Acupuncture can effectively improve the behavioral performance of rates with migraine, and its mechanism of action might involve the inhibition of meningeal vasodilation and inflammatory factors, and the reduction of neurogenic inflammation.

## 1 Introduction

Migraine is a common primary headache, characterized by recurrent unilateral or bilateral throbbing severe headache accompanied by nausea and photophobia [1]. The underlying etiology of the disease is due to disturbances in nerve cell excitability, genetic, endocrine and metabolic factors, and is easily triggered by insomnia, stress, exertion, noise, diet, weather and menstrual cycle, etc. The pathogenesis of migraine has not been fully elucidated in Western medicine, and the mainstream is the trigeminal vascular inflammatory theory [2]. This theory suggests that the main physiopathological changes that occur in migraine are the dilation of meningeal blood vessels and the release of inflammatory factors leading to a neurogenic inflammatory response, which results in headache symptoms. At present, migraine is mainly treated with analgesic and sedative drugs. Although their immediate efficacy is positive, their safety, tolerability and long-term curative effects are not satisfactory [3]. Therefore, the active exploration of effective and low-risk treatment methods is the current focus of migraine prevention and treatment.

Migraine is the preponderant illness for therapy in Chinese acupuncture. Studies have shown that acupuncture can reduce migraine frequency, pain degree and duration, with low side effects [4,10]. To date, there is a substantial body of animal experiments that demonstrated better therapeutic outcomes of acupuncture in the treatment of migraine, but the relevant systematic evaluation of its effects is limited.

The objective of our systematic review and meta-analysis is to explore the efficacy of acupuncture and to discuss the potential working mechanisms of acupuncture in protecting against migraine. Results obtained from this analysis may provide the therapeutic basis of acupuncture in the treatment of migraine in humans.

## 2 Materials and methods

### 2.1 Eligibility criteria

Inclusion criteria: (1) Research type: aniaml studies published in Chinese and English; (2) Research subject: experimental migraine model in rats with nitroglycerin; (3) Intervention study: the control group was migraine model group, the experimental group was migraine rat model treated with acupuncture. The difference of intervention measures between the control and experimental groups should only be whether acupuncture treatment was used. (4) Efficacy evaluation index: behavioral scores, calcitonin gene-related peptide (CGRP), substance P (SP), 50% paw withdrawal threshold (50%PWT), stimulatory G protein (Gsa), inhibitory G protein (Gia) and nitric oxide (NO).

Exclusion criteria: (1) Research type: non-animal experiments, reviews, case reports, etc.; (2) Research subject: non-nitroglycerin-induced migraine models such as electrical stimulation; (3) Insufficient data; (4) Inconsistent outcome measures.

### 2.2 Study strategy

We selected relevant studies published by June 2022 through searching databases including Chinese National Knowledge Infrastructure (CNKI), and Wanfang Data information site, vip Database (VIP), Chinese Biomedicine Database (CBM), PubMed, Cochrane Library, Web of Science and EBSCO. Search terms were: (Acupuncture OR Pinprick OR Moxibustion OR Acupoint) AND (Disorder Migraine OR Disorders Migraine OR Migraine Disorder OR Cerebrovascular Accident OR Cerebrovascular Accidents OR CVA Cerebrovascular Migraine OR Migraines OR Migraine Headache OR Headache Migraine OR Headaches Migraine). Comprehensive and systematic retrieval were conducted according to different database situations.

### 2.3 Data extraction

Two researchers with the same training independently screened and extracted the data, and any disagreement was resolved by the third researcher. The following data were extracted from the selected studies: (1) General information including the title, author, year, the animal quantity, weight, species and model; (2) Intervention including acupuncture point, acupuncture manipulation method and acupuncture duration; (3) Outcomes. For the literature that only reported data in the form of images, we extracted data from the images through GetData Graph Digitizer software; (4) Adverse reactions.

### 2.4 Quality assessment

Statistical software SYRCLE’s Risk of Bias tool [5] was used to make judgments for each entry.

### 2.5 Data analysis

Statistical software Revman 5.3 was used to analyze the collected data. Counting data were expressed as relative risk (RR) and its 95% confidence interval (CI), and continuous variables were represented as mean difference (MD) or standardized mean difference (SMD) that was to calculate 95% confidence interval (95% CI). When interventions are measured in identical methods or units, MD was used. When they were different, SMD was used. When heterogeneity test results were *P*>0.05 and I^2^<50%, the fixed effect models were used. When the heterogeneity test results were *P*<0.05 and I^2^>50%, the random effect models were used. In order to find out the sources of heterogeneity, sensitivity analysis or subgroup analysis were performed. For the literature with excessive heterogeneity, the final results were described and analyzed by forest and funnel maps.

## 3 Results

### 3.1 Inclusion of literature analysis

**Fig 1:**
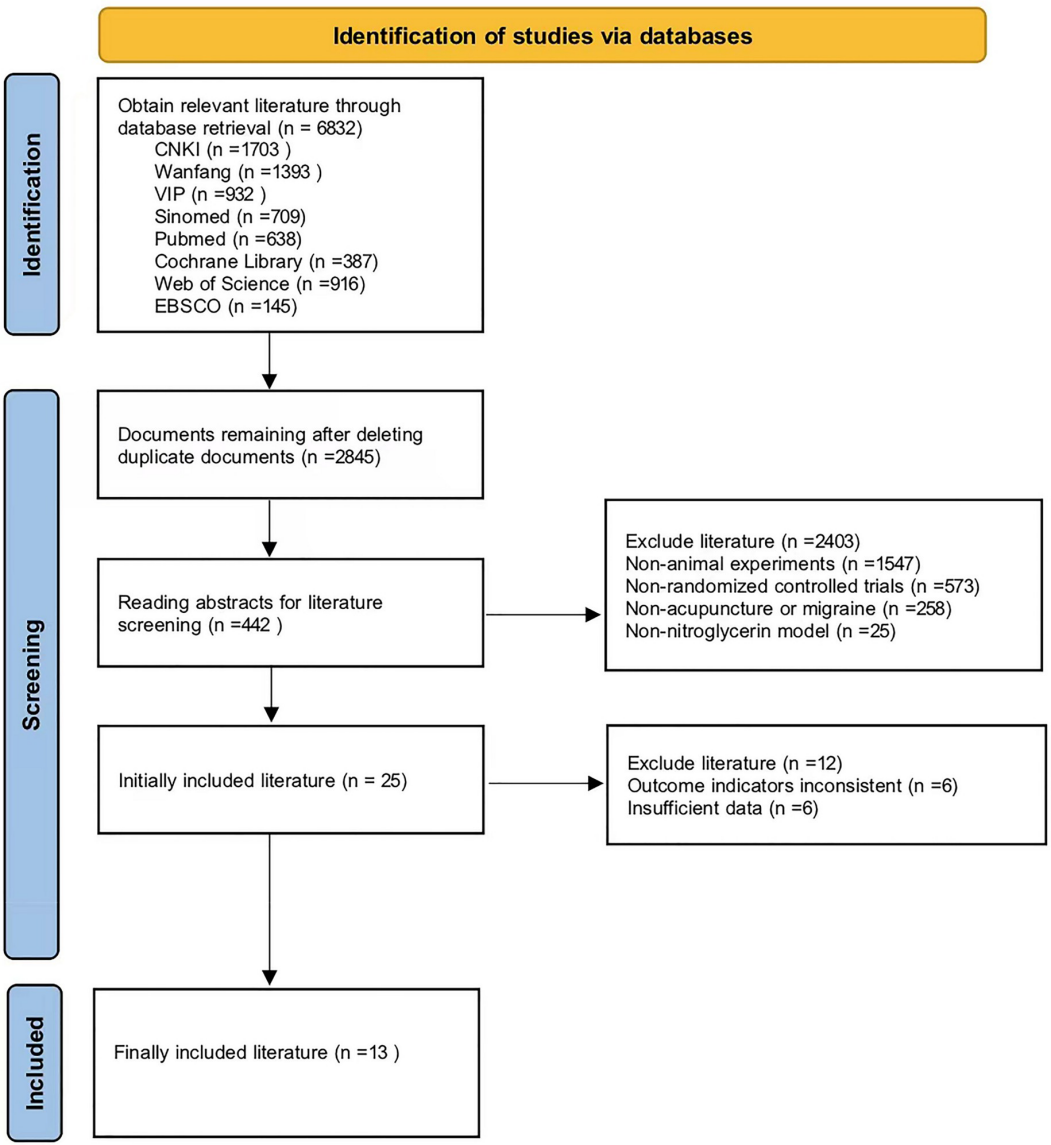
Flow diagram of literature screening.

We identified a total of 6832 literatures, 2845 studies remained after excluding duplicate articles, and 442 studies remained after excluding non-migraine, non-acupuncture, and non-nitroglycerin models. Eventually, 13 studies were included in the final meta-analysis (Fig 1). The basic characteristics of the included studies were shown in Table 1. The risk of bias for the included studies were shown in Table 2.

**Table 1:** Basic characteristics of included studies.

Included study	Animal characteristics	Animal models	Animal numbers (E/C)	Intervention measures			Outcome measures	Adverse reactions
				Acupoint	Manipulation	Time		
Song, Boqi 2021 [6]	SD rats(225±25) g, half male and half female	Nitroglycerin	10/10	Fengchi(g20),Waiguan(te5),Yanglingquan(g34)	even reinforcing-reducing method, twirling180°, 120/min	manipulating needle 0.5~2min, retaining needle 30min	①②	Not mentioned
Zhang, Yalan 2021 [7]	SD rats(225±25) g, half male and half female	Nitroglycerin	10/10	Fengchi(g20),Waiguan(te5), Yanglingquan(g34)	even reinforcing-reducing method, twirling180°, 120/min	manipulating needle 0.5~2min, retaining needle 30min	①②	Not mentioned
Huang, shaolei2021 [8]	Wistar rats(200–240) g, male	Nitroglycerin	8/8	Fengchi(g20), Baihui(gv20), Taichong(liv3), Neiguan(p6)	no report	retaining needle 30min	①③④	Not mentioned
Huang, shaolei2020 [9]	Wistar rats(200–240) g, male	Nitroglycerin	8/8	Fengchi(g20), Baihui(gv20), Taichong(liv3), Neiguan(p6)	no report	retaining needle 30min	①	Not mentioned
Wang, Qianna2019 [10]	SD rats(200–250) g, no mention of gender	Nitroglycerin	10/10	Fengchi(g20),Waiguan(te5),Yanglingquan(g34)	even reinforcing-reducing method, twirling180°, 120/min, electropuncture (10/50Hz)	manipulating needle 0.5~2min, retaining needle 30min	①②③	Not mentioned
Wang,Mengmeng 2018 [11]	Wistar rats(200–240) g, male	Nitroglycerin	8/8	Baihui(gv20), Fengchi(g20), Neiguan(p6), Taichong(liv3)	even reinforcing-reducing method, low amplitude twirling	manipulating needle per 10min, retaining needle 30min	①③④	not mentioned
Ye, Yihong 2018 [12]	SD rats(200–250) g, half male and half female	Nitroglycerin	10/10	Waiguan(te5), Yanglingquan(g34)	even reinforcing-reducing method, twirling180°, 120/min, electroacupuncture (10/50Hz)	manipulating needle 0.5~2min, retaining needle 30min	②⑦	not mentioned
Meng, Xianhui 2014 [13]	SD rats(180–290) g, half male and half female	Nitroglycerin	10/10	Fengchi(g20), Zhongzhu(te3),Waiguan(te5)	no report	retaining needle 30min	④	not mentioned
Zhao, Zhien 2013 [14]	Rats, no mention of gender	Nitroglycerin	12/12	Shuaigu(g8), Jiaosun(te20), Hanyan(g4), Sanyangluo(te8), yangfu(g38), Taichong(liv3)	even reinforcing-reducing method	manipulating needle per 10min, retaining needle 20min	⑦	not mentioned
Li, Lihong 2011 [15]	SD rats(220–240) g, female	Nitroglycerin	12/12	Guanyuan(cv4), Zusanli(s36), Shenshu(b23), Sanyinjiao(sp6)	no report	retaining needle 20min	③	not mentioned
Li, Zhenyan 2005 [16]	SD rats (270±20) g, female	Nitroglycerin	10/10	Yanglingquan(g34), Fengchi(g20)	no report	retaining needle 20min	⑤⑥	not mentioned
Wang Su’e 2005 [17]	SD rats (270±20), female	Nitroglycerin	10/10	Waiguan(te5), Yifeng(te17)	no report	retaining needle 20min	⑤⑥	not mentioned
Zhong, Guangwei 2004 [18]	Wistar rats (270±20) g, female	Nitroglycerin	10/10	Taichong(liv3),Ququan(liv8)	electroacupuncture (20/40Hz)	retaining needle 20min	⑤⑥	not mentioned

**Note:** E: Experimental group; C: Control group; ①behavioral scores; ②50%PWT; ③SP; ④CGRP; ⑤Gsa; ⑥Gia; ⑦NO.

**Table 2:** Methodologic quality assessment of the risk of bias.

Included study	①	②	③	④	⑤	⑥	⑦	⑧	⑨	⑩
Song, Boqi 2021 [6]	Y	Y	U	U	U	U	U	U	Y	N
Zhang, Yalan 2021 [7]	Y	Y	U	U	U	U	U	U	Y	N
Huang, shaolei2021 [8]	Y	Y	U	U	U	U	U	U	Y	N
Huang, shaolei2020 [9]	Y	Y	U	U	U	U	U	U	Y	N
Wang, Qianna2019 [10]	Y	Y	U	U	U	U	U	U	Y	U
Wang, Mengmeng 2018 [11]	Y	Y	U	U	U	U	U	U	Y	N
Ye, Yihong 2018 [12]	Y	Y	U	U	U	U	U	U	N	U
Meng, Xianhui 2014 [13]	Y	Y	U	U	U	U	U	U	Y	U
Zhao, Zhien 2013 [14]	U	Y	U	U	U	U	U	U	Y	U
Li, Lihong 2011 [15]	U	Y	U	U	U	U	U	U	Y	U
Li, Zhenyan 2005 [16]	U	Y	U	U	U	U	U	U	Y	U
Wang Su’e 2005 [17]	U	Y	U	U	U	U	U	U	Y	U
Zhong, Guangwei 2004 [18]	Y	Y	U	U	U	U	U	U	Y	U

**Note:** Y: Low risk of bias; N: High risk of bias; U: Uncertain risk of bias; ①sequence generation; ②baseline characteristics; ③allocation concealment; ④randomization of animal placement; ⑤researcher blinding; ⑥evaluation of random results; ⑦tester blinding; ⑧incomplete data reporting; ⑨selective results reporting; ⑩other bias.

### 3.2 Behavioral scores analysis

Six studies [6–11] reported behavioral scores. Meta-analysis of these studies showed that there was heterogeneity (I^2^ = 88%, *P*<0.00001), so the random effect model was adopted. The results showed that acupuncture could effectively reduce behavioral scores (MD = -15.01, 95%CI = [-18.01, -12.01], *P*<0.00001), and the difference was statistically significant (Table 3).

**Table 3:** Meta-analysis results of each outcome indicator.

outcome indicator	Heterogeneity test results		Effect models	Meta-analysis results		
	I^2^ (%)	*P*		Effect sizes	95%CI	*P*
Behavioral scores	88	<0.00001	Random	MD = -15.01	[-18.01, -12.01]	<0.00001
CGRP	97	<0.00001	Random	MD = -16.14	[-21.45, -10.83]	<0.00001
SP	96	<0.00001	Random	MD = -11.47	[-15.97, -6.98]	<0.00001
50%PTW	87	<0.0001	Random	MD = 1.96	[1.15, 2.77]	<0.00001
Gsa	0	0.50	Fixed	MD = 24.01	[20.10, 27.92]	<0.00001
Gia	0	0.50	Fixed	MD = 24.01	[20.10, 27.92]	<0.00001
NO	0	0.99	Fixed	MD = -3.02	[-3.79, -2.26]	<0.00001

### 3.3 CGRP analysis

Three studies [8,11,13] reported CGRP and showed relatively high heterogeneity (*P*<0.00001, I^2^ = 97%), and the random effect model was used. The results showed that acupuncture could reduce the content of CGRP in serum (MD = -16.14, 95%CI = [-21.45, -10.83], *P*<0.00001), and the difference was statistically significant (Table 3).

### 3.4 SP analysis

Four studies [8,10,11,15] reported SP. The study showed that the heterogeneity was relatively large (*P*<0.00001, I^2^ = 96%), and the random effect model was used. The results showed that acupuncture was effective and could significantly reduce the content of SP in serum (MD = -11.47, 95%CI = [-15.97, -6.98], *P*<0.00001), and the difference was statistically significant (Table 3).

### 3.5 50%PTW analysis

Four studies [6,7,10,12] reported 50%PTW. Meta-analysis of these studies indicated that there was heterogeneity (*P*<0.0001, I^2^ = 87%), so the random effect model was adopted. The results showed that acupuncture could obviously improve 50% PTW (MD = 1.96, 95%CI = [1.15, 2.77], *P*<0.00001), and the difference was statistically significant (Table 3).

### 3.6 Gsa analysis

Three studies [16–18] reported Gsa. The forest plot showed that there was heterogeneity (*P* = 0.87, I^2^ = 0%), so the fixed effect model was adopted. The results showed that acupuncture could significantly reduce the content of Gsa in brainstem (MD = -62.90, 95%CI = [-69.88, -55.92], *P*<0.00001), and the difference was statistically significant (Table 3).

### 3.7 Gia analysis

Three studies [16–18] reported Gia. They showed that there was heterogeneity (*P* = 0.50, I^2^ = 0%), so the fixed effect model was adopted. The results showed that acupuncture could significantly increase the content of Gia in brainstem (MD = 24.01, 95%CI = [20.10, 27.92], *P*<0.00001), and the difference was statistically significant (Table 3).

### 3.8 NO analysis

Two studies [12,14] reported NO. They showed that there was heterogeneity (*P* = 0.99, I^2^ = 0%), so the fixed effect model was adopted. The results showed that acupuncture could effectively reduce the content of NO in serum (MD = -3.02, 95%CI = [-3.79, -2.26], *P*<0.00001), and the difference was statistically significant (Table 3).

### 3.9 Sensitivity analysis

#### 3.9.1 Behavioral scores

**Fig 2:**
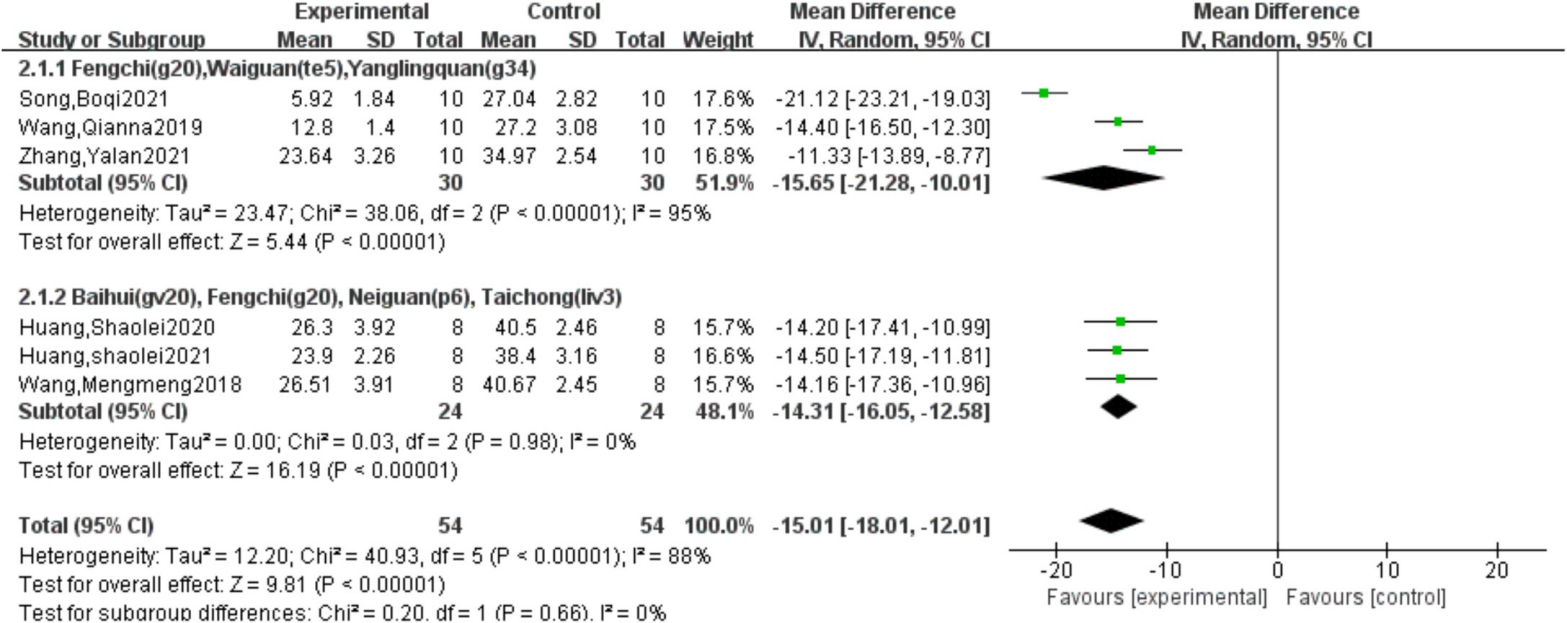
Subgroup analysis of behavioral scores.

Subgroup analysis was performed using acupoints as grouping criteria, and the results of the heterogeneity test showed that the subgroup I^2^ increased to 95% for acupoints Fengchi, Waiguan, and Yanglingquan, and decreased to 0% for acupoints Baihui, Fengchi, Neiguan, and Taichong, indicating that the different acupoints had an effect on the heterogeneity of this study. However, the results still support that acupuncture is effective in reducing behavioral scores and improving behavioral performance in migraine rats (Fig 2).

#### 3.9.2 CGRP

We conducted the sensitivity analysis of CGRP by separately leaving each one study out. Study reported by Meng, Xianhui [13] is the main source of heterogeneity, and I^2^ after excluding the study by Meng, Xianhui [13] was reduced from 97% to 0%. Due to the limited studies included in the meta-analysis, we speculated that the method of CGRP measurement in the study was radioimmunoassay [13], while others were ELISA [8,11]. Differences in the methods of CGRP measurements might lead to heterogeneity. However, the results still support that acupuncture can reduce the content of CGRP in serum of rats with migraine.

#### 3.9.3 SP

**Fig 3:**
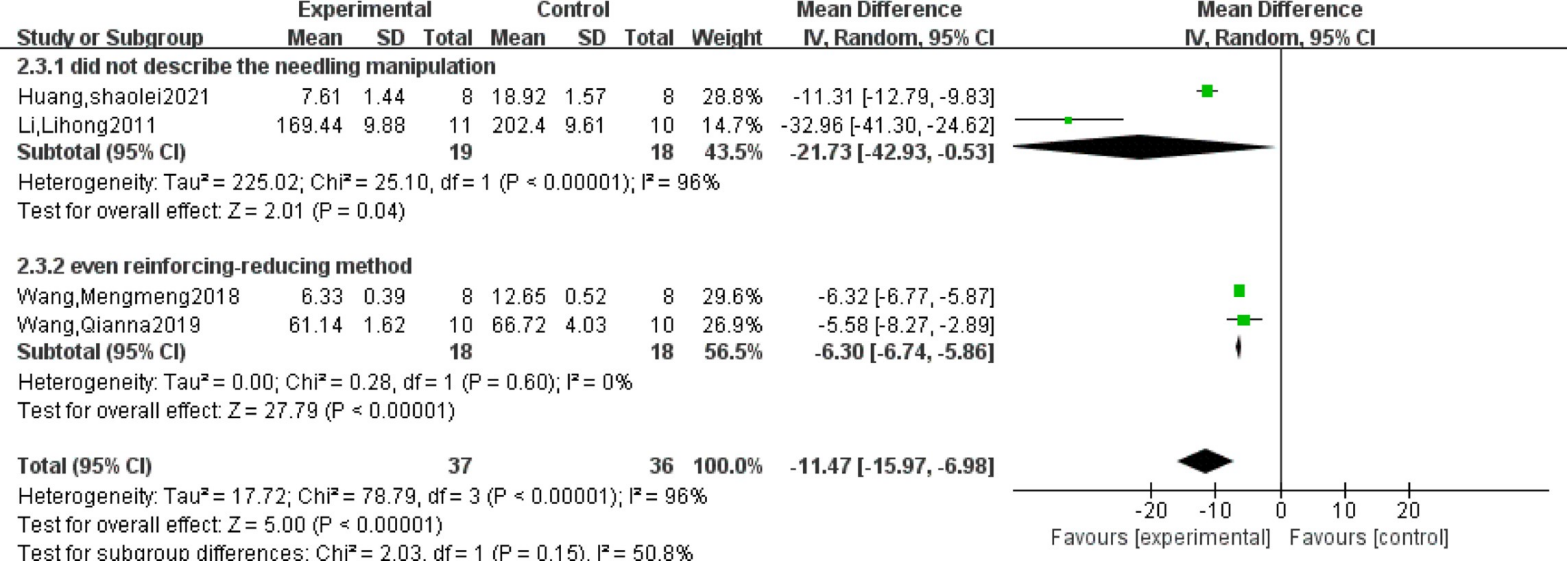
Subgroup analysis of SP.

The subgroup analysis of SP showed that I^2^ increased to 96% in the subgroup that did not describe the acupuncture manipulation. I^2^ decreased to 0% in the subgroup that used the reinforcing-reducing method. Fig 3 shows that difference in acupuncture manipulation is the main cause of heterogeneity of SP. The results also support that acupuncture was effective in reducing SP in serum of rats with migraine.

#### 3.9.4 50%PTW

**Fig 4:**
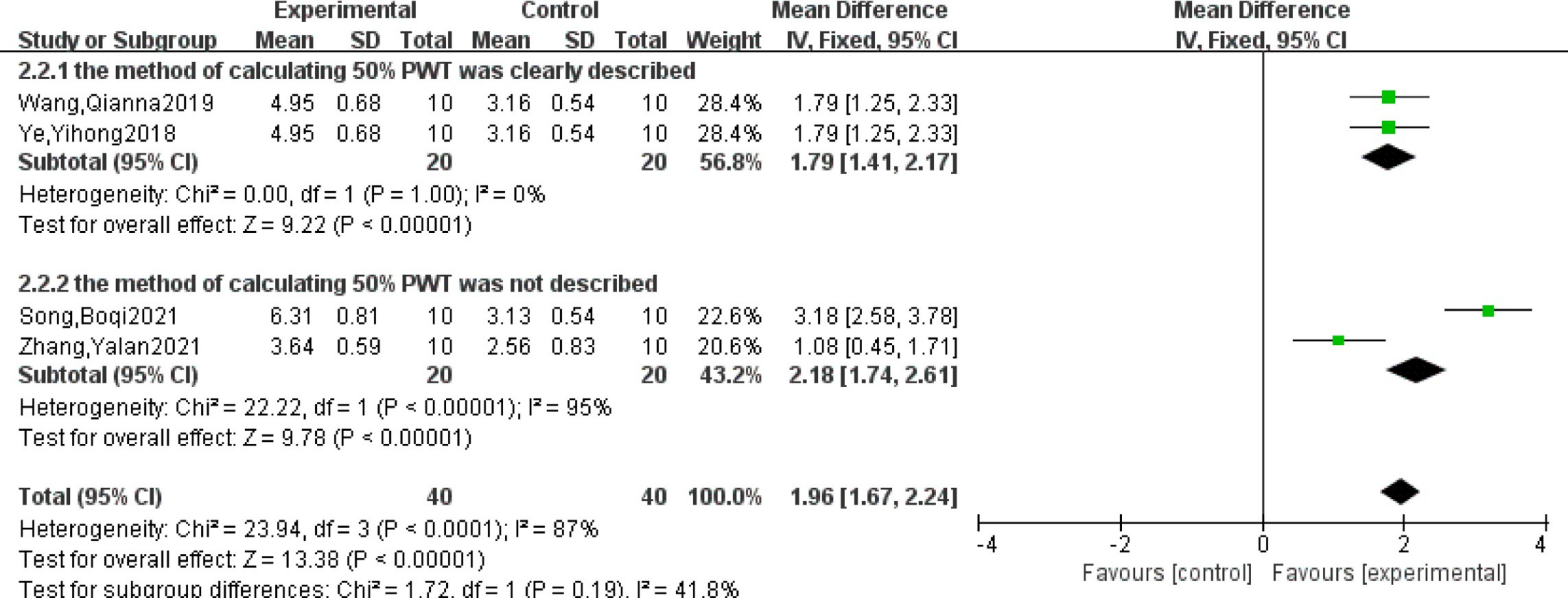
Subgroup analysis of 50%PTW.

The subgroup analysis of 50%PWT showed that I^2^ decreased to 0% in the subgroup that the method of calculating 50%PWT was clearly described, and I^2^ increased to 95% in those that the method of calculating 50%PWT was not described. Fig 4 shows different methods calculating of 50%PWT that are the main cause of heterogeneity. The results also support that acupuncture was effective in increasing 50%PTW, thereby improving pain sensitivity in rats with migraine.

### 3.10 Publication bias

**Fig 5:**
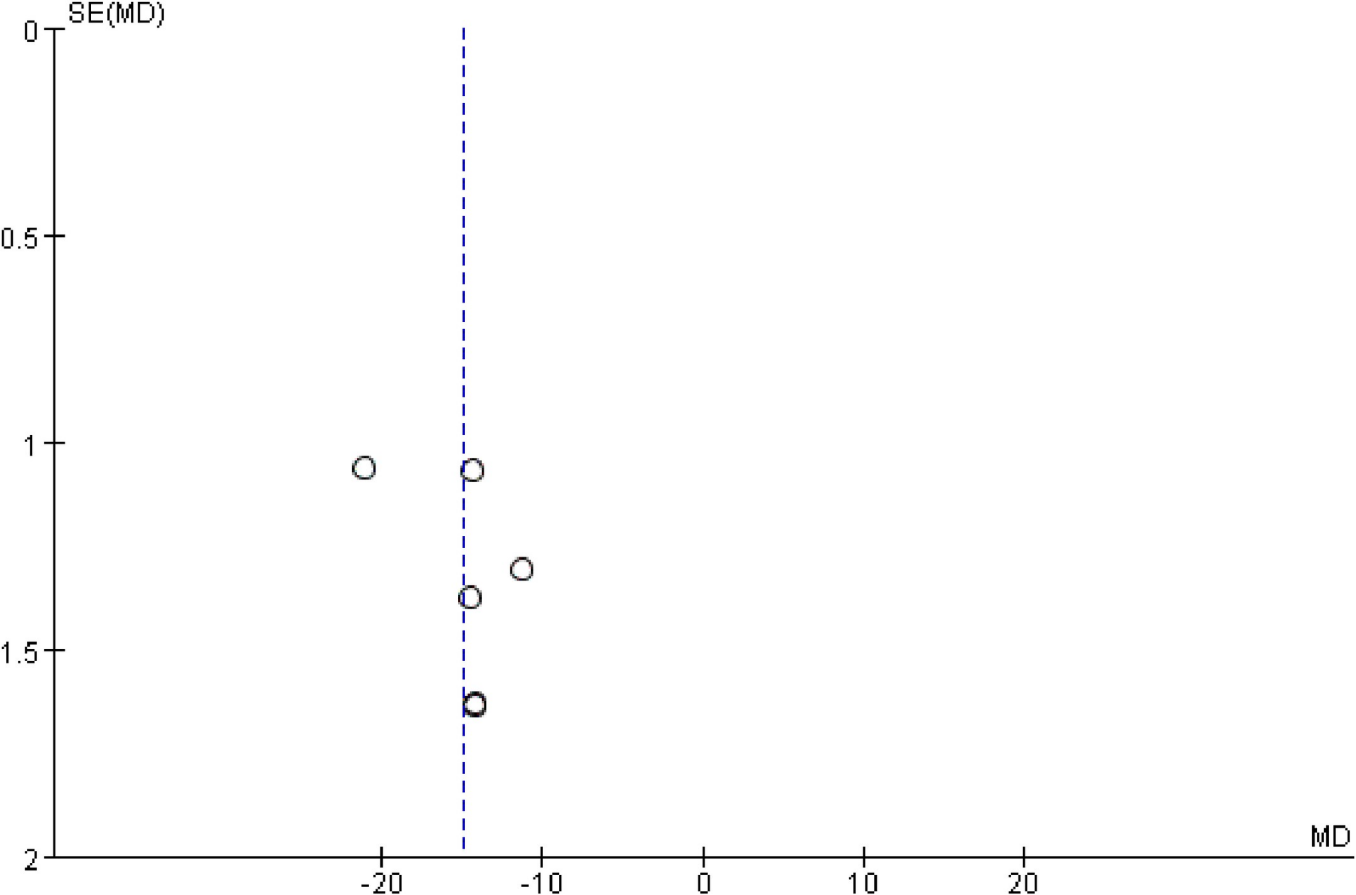
Funnel plot of behavioral scores.

The publication bias of behavioral scores was assessed by the funnel plot, as shown in Fig 5. The results showed a symmetrical trend and some publication bias.

## 4 Discussion

Migraine is commonly encountered in clinical practice. In recent years, acupuncture has been used widely to treat patients with migraine and other pain conditions. In order to better understand the clinical effect of acupuncture on migraine, we conducted a meta-analysis that included randomized, controlled experimental studies in rat models of migraine. This study is the first systematic review of acupuncture treatment of migraine rats. We demonstrated that acupuncture attenuates migraine in rats and that its beneficial effect associated with the reduction of CGRP, SP, Gsa, Gia, and NO, thus improving behavioral scores and 50%PWT.

CGRP plays a pivotal role in the pathogenesis of migraine [19]. In the peripheral trigeminal vascular system, CGRP is released and participates in the activation of meningeal injury receptors. CGRP can enhance pain-sensory transduction by activating mast cells, immune cells and endothelial cells, leading to dilation of intracranial arteries [20]. SP participates in the regulation of pain perception, which can both cause pain and relieve pain in the nervous system. SP can stimulate the secretion of inflammatory factors by binding to the specific receptor, resulting in increased nerve cell excitation and sensitivity that induce neuroendocrine and neuroinflammation. CGRP and SP coexist in sensory ganglion nerve cells. SP interacts with CGRP promoting the secretion of SP, which in turn strengthens the transmission of pain signals. G protein plays an important role in intra- and extra-cellular signal transduction systems. Both Gsa and Gia are closely related to cerebral vasomotor function [21]. In the neurovascular endothelial cell receptor-adenylase (AC) signaling system, Gsa activates AC and converts adenylate into cAMP, while Gia inhibits AC activity and affects cAMP production, thus regulating the release of vasoactive substances and neuropeptides in the brain. Acupuncture treatment can effectively reduce the content of Gsa and increase the content of Gia, thereby reducing the production of cAMP and improving the intracranial vasodilatory function. NO plays a key role in migraine by regulating the diastolic function of brain microvascular endothelial cells. NO can not only mediate endothelium-dependent relaxation, but also mediate cerebral vascular dilation caused by local neuronal excitation [22]. NO can cause the onset of pain sensation by directly affecting paravascular sensory nerves to activate sensory nerve fibers, improving the sensitivity of nociceptive sensory neurons, mediating the transmission of pain signals [23]. NO exerts migraine inducing effect by upregulating inflammatory cytokines such as interleukin-1 and tmor necrosis factor-α that stimulate trigeminal nerve fibers to promote the release of CGRP, SP and other vasoactive substances [24].

**Fig 6:**
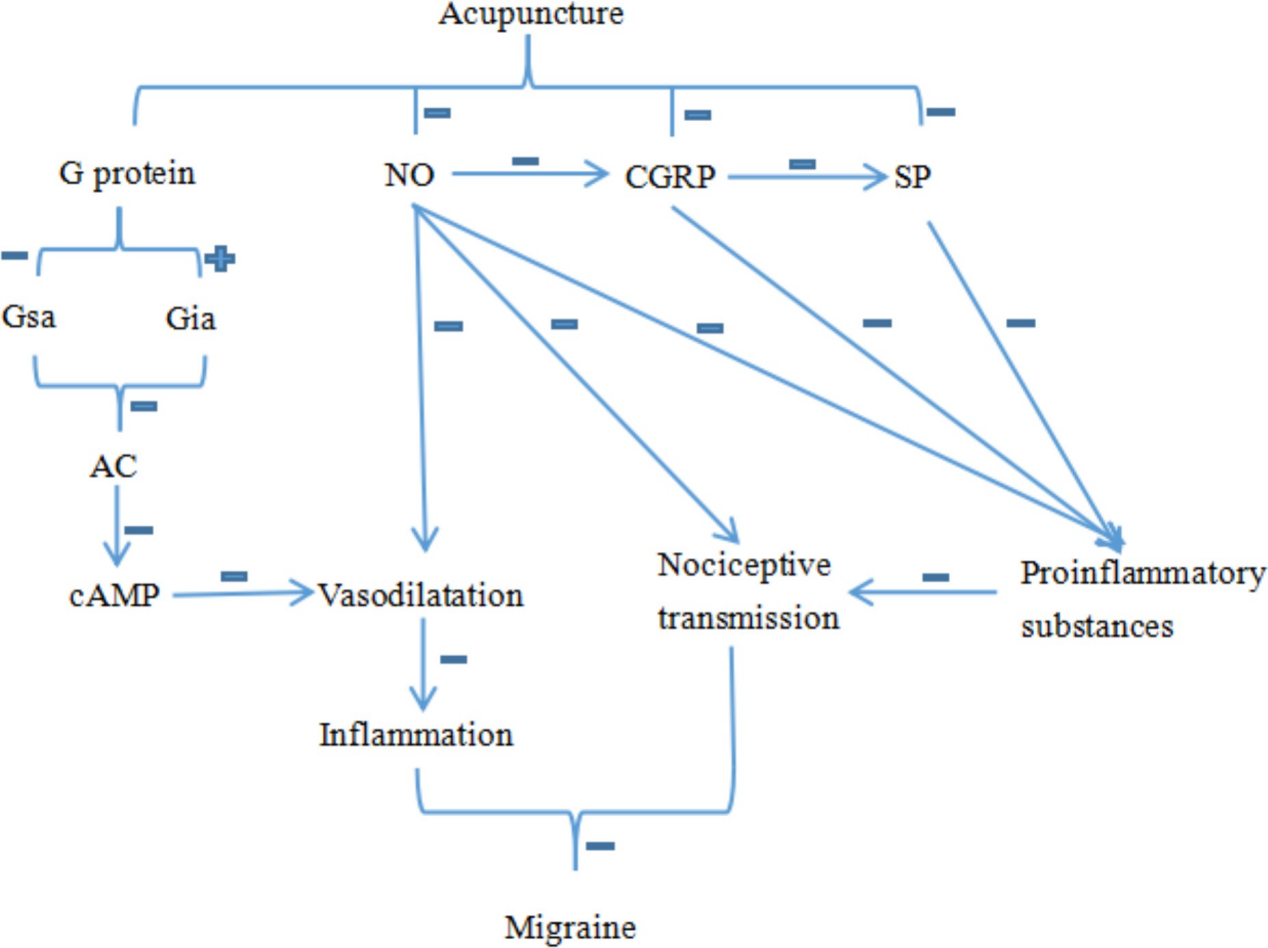
Mechanism of acupuncture in the treatment of migraine.

As shown in the meta-analysis, the levels of GCRP, SP and NO were increased in migraine models in rats, and the increase of these neuroinflammatory mediators were attenuated by acupuncture treatment. Based on the current systematic review and mete-analysis, acupuncture protects against migraine in rats was associated with elevated the 50%PTW value, decreased levels of Gsα, increased the level of Giα, and reduced Gsα/Giα protein ratio, thus regulating the signaling system inside and outside the cell membrane and the transcriptional expression of various target genes in the nucleus, leading to the improvement of the intracranial vasomotor function (Fig 6).

The limitations of this system review included: (1) The studies included in the data analysis were limited, which might have a certain bias on the conclusion. (2) There is a risk of uncertainty bias in multiple evaluation items, suggesting that randomized controlled trials with standardized experimental designs should be selected in the future to exclude the implementation bias and measurement bias in the study.

## 5 Conclusion

In conclusion, this systematic review and meta-analysis demonstrated that acupuncture attenuates and prevents migraine in rats, compared with the sham acupuncture group. This study illustrates that the effectiveness of acupuncture treatment in migraine is mainly by alleviating neurogenic inflammation and central nociceptive conduction via the regulation of meningeal vasodilation and inflammatory factors, providing a therapeutic basis for acupuncture in the treatment of migraine. However, potential publication bias still exists. In the future, high-quality trials are needed to minimize bias, thus proving more persuasive findings that can be used to improve migraine patient care.

## Supporting information

S1 File PRISMA 2020 checklist. (DOCX)
